# Primary Health Care: care coordinator in regionalized networks?

**DOI:** 10.1590/S1518-8787.2016050006602

**Published:** 2016-11-24

**Authors:** Patty Fidelis de Almeida, Adriano Maia dos Santos

**Affiliations:** I Departamento de Planejamento em Saúde. Instituto de Saúde Coletiva. Universidade Federal Fluminense. Niterói, RJ, Brasil; II Instituto Multidisciplinar em Saúde. Campus Anísio Teixeira. Universidade Federal da Bahia. Vitória da Conquista, BA, Brasil

**Keywords:** Primary Health Care, organization & administration, Health Services, supply & distribution, Health Services Coverage, Regional Health Planning, Systems Integration

## Abstract

**OBJECTIVE:**

To analyze the breadth of care coordination by Primary Health Care in three health regions.

**METHODS:**

This is a quantitative and qualitative case study. Thirty-one semi-structured interviews with municipal, regional and state managers were carried out, besides a cross-sectional survey with the administration of questionnaires to physicians (74), nurses (127), and a representative sample of users (1,590) of *Estratégia Saúde da Família* (Family Health Strategy) in three municipal centers of health regions in the state of Bahia.

**RESULTS:**

Primary Health Care as first contact of preference faced strong competition from hospital outpatient and emergency services outside the network. Issues related to access to and provision of specialized care were aggravated by dependence on the private sector in the regions, despite progress observed in institutionalizing flows starting out from Primary Health Care. The counter-referral system was deficient and interprofessional communication was scarce, especially concerning services provided by the contracted network.

**CONCLUSIONS:**

Coordination capacity is affected both by the fragmentation of the regional network and intrinsic problems in Primary Health Care, which poorly supported in its essential attributes. Although the health regions have common problems, Primary Health Care remains a subject confined to municipal boundaries.

## RESUMO

**OBJETIVO:**

Analisar o alcance da coordenação do cuidado pela Atenção Primária à Saúde em três regiões de saúde.

**MÉTODOS:**

Trata-se de estudo de caso, com abordagem quantitativa e qualitativa. Foram realizadas 31 entrevistas semiestruturadas com gestores municipais, regionais e estaduais e estudo transversal com aplicação de questionários para médicos (74), enfermeiros (127) e amostra representativa de usuários (1.590) da Estratégia Saúde da Família em três municípios-sede de regiões de saúde do estado da Bahia.

**RESULTADOS:**

A função de porta de entrada preferencial pela Atenção Primária à Saúde deparava-se com forte concorrência de serviços ambulatoriais hospitalares e de pronto-atendimento, desarticulados da rede. Problemas de acesso e oferta de atenção especializada eram agravados pela dependência do setor privado nas regiões, ainda que tenham sido observados avanços na institucionalização de fluxos desde a Atenção Primária à Saúde. A contrarreferência era deficiente e a comunicação interprofissional escassa, principalmente quando o usuário era atendido na rede contratada ou conveniada.

**CONCLUSÕES:**

A capacidade de coordenação mostra-se afetada tanto pela fragmentação da rede regional, quanto por problemas intrínsecos à Atenção Primária à Saúde, pouco fortalecida em seus atributos essenciais. Apesar de as regiões de saúde apresentarem problemas em comum, a Atenção Primária à Saúde continua sendo um tema circunscrito aos limites municipais.

## INTRODUCTION

Lack of care coordination is identified as a major cause of poor quality in health services, associated with higher costs, duplication and overuse of diagnostic procedures, use of multiple medicines, and conflicting therapies, with chronic conditions suffering the greatest negative impact^6^ ^,^ ^12^ ^,^ ^14^ ^,^ ^a^.

Coordination presupposes organizing patient care, which may involve two or more providers and users themselves, in order to facilitate the timely provision of services, involving planning related to staff and other resources and instruments for information exchange between providers^13^ ^,^ ^17^.

Organizational elements to ensure coordination should include the definition of shared goals for the health system; financial incentives via disbursement and allocation of resources; communication mechanisms between health professionals; development of a common culture and leadership oriented towards teamwork, collaboration and better performance; and strengthening of a care model based on Primary Health Care (PHC)^a^.

Different contexts must be considered, since there is no broadly developed definition^13^. The *Política Nacional de Atenção Básica* (PNAB – National Primary Care Policy)^b^ defines coordination as “coordination of integrality,” one of the basis of PHC, which should be enabled by means of horizontal integration strategies (programmatic action and spontaneous demand, surveillance and care initiatives, multidisciplinary and interdisciplinary work) and vertical integration strategies between different levels of the Healthcare Networks.

In Brazil, studies suggest that integration of Healthcare Networks, one of the dimensions of coordination, has been strengthened by expansion of Family Health Strategy; creation of specialized services in health districts; introduction of a regulatory system; computerization of medical records; development of management and clinical protocols; and initiatives in communication and technical support^1^ ^,^ ^8^ ^,^ ^9^ ^,^ ^15^.

In this article, care coordination is understood as interaction between various services, actions and professionals related to health care to ensure it is always synchronized and focused on achieving a common goal, regardless of where it is provided^11^. It is supported by the existence of integrated action between providers at different levels or within a same level, so that different interventions are perceived and experienced by users in a manner that is continuous and appropriate to their health needs^5^.

This article sought to examine the breadth of care coordination by PHC in regionalized networks. It aims to contribute new elements by debating the issue in the context of health regions, given the lack of research proposing to investigate coordination in circumstances that require horizontal integration between same-level professionals and services providers, based on PHC with strong essential attributes, and vertical integration between network services managed by different state agencies. Therefore, analyzing facilitating devices and barriers to coordination in regional areas may indicate paths to achieve timelier and higher quality access to the Brazilian Unified Health System (SUS).

## METHODS

This is a case study carried out in three municipal centers of the health regions of Feira de Santana, Santo Antônio de Jesus and Vitória da Conquista, in the state of Bahia. It combined qualitative and quantitative approaches – “mixed methods”^10^, using semi-structured interviews and surveys.

The qualitative analysis was performed based on 31 semi-structured interviews with managers at municipal, state and regional level, in 2012.

The experiences of professionals were assessed based on self-administered questionnaires with physicians and nurses of *Equipes de Saúde da Família* (EqSF – Family Health Teams) in activity in October 2012: 84 in Feira de Santana, 21 in Santo Antônio de Jesus and 38 in Vitória da Conquista, with estimated population coverage of 51.5%, 78.7% and 41.2%, respectively^c^. Physicians and nurses were chosen because they are more directly involved in coordinating activities. The instrument used was adapted from a study by Giovanella et al.^d^ (2008). The survey administered 106 questionnaires in Feira de Santana, 31 in Santo Antônio de Jesus, and 64 in Vitória da Conquista, i.e., 201 of the 286 planned questionnaires. Losses (30%) are unevenly distributed between physicians (48%) and nurses (11%), justified not by refusal, but by precarious labor contracts, deteriorated during the period of municipal elections.

A household-based survey was carried out, with a questionnaire administered to a representative sample of families enrolled in EqSF in each municipality. The family respondent was the head of household or spouse, interviewed at home. The questionnaire for users was also adapted from Giovanella et al.^c^ Calculation of the users sample considered the percentage of population covered by EqSF (*p*), according to *Caderno de Avaliação e Monitoramento da Atenção Básica* ^e^ (Primary Care Monitoring and Assessment Register), assuming 95% confidence level (*z* ^2^) (represented by the value 1.96, of the normal distribution abscissa [0.1]) and 4% accuracy (*w –* π)^2^. The number of users, assuming a simple random sample, was 596, 430 and 576 for Feira de Santana, Santo Antônio de Jesus and Vitória da Conquista, respectively, for a total sample of 1,602 users.

Users were selected by cluster sampling in three stages. The first selected the number of users to be interviewed in each EqSF, dividing the sample of each municipality by the number of existing teams in October 2012. In the second stage, a community health worker was selected for each EqSF by simple random sampling. Finally, the number of families of the selected community health worker was drawn. The questionnaires for professionals and users were administered between November 2012 and February 2013. For users, 1,590 questionnaires were administered.

The concept of coordination used in the study was operationalized by means of dimensions and indicators that incorporate components to strengthen the essential attributes of PHC, such as organization as first contact of preference and breadth/resolvability; and integration of Healthcare Networks, such as provision of and access to specialized care and communication between professionals (Table 1).

**Table 1:** Matrix structure for analysis of care coordination by Primary Health Care in Health Regions.

Dimension	Indicator
Organization of PHC as first contact of preference	Awareness of ESF (U) Awareness of and easy access to ESF facility (U) Knowledge of and home visit by CHW (U) Private health insurance coverage (U) USF as regularly sought service and first contact (U/P) Average waiting time for medical appointment at USF (P) Scheduling medical appointments (U) Spontaneous demand care (U/P) Regional strategies to strengthen PHC (M)
PHC breadth and resolvability	Strategies to increase PHC breadth and resolvability (techincal support, oral health coverage, physical infrastructure and facilities, logistics and communication support) (M) Availability of physicians (M) EqSF professional responsible for care (U) Satisfaction with care (U) Resolution of health problem in EqSF care (U/P) Actions executed when health problem is not resolved (U) Sample collection for lab tests at USF (P) Performance of clinical pathology tests required by EqSF (U) Access to medicines prescribed by EqSF (U/P)
Provision of and access to specialized care in Healthcare Network	Adequate provision of specialized care in HN (M) Provision of specialized care appointments (U) Forms of access to specialized care appointments referred by EqSF (U/P/M) Scheduling of specialized care appointments and tests and hospitalization by EqSF (P) Performance of specialized lab tests requested by EqSF (U) Forms of access to specialized lab tests (U) Main characteristic of specialized services (M) Regional strategies to provide specialized care (M)
Communication between professionals	Use of referral and counter-referral instruments (P/M) Computerization of health services (M) Use of clinical protocols (M) Completion of medical records following appointments at USF (P) Follow-up care of users of other health services (U/P)

U: users/families; P: professionals (physicians and nurses) of family health teams; M: health managers; ESF: Family Health Strategy; USF: Family Health Unit; CIR: *Comissão Intergestores Regional* (Regional Inter-Managerial Commission); CHW: Community Health Worker; EqSF: *Equipe de Saúde da Família* (Family Health Team); HN: Healthcare Networks

The qualitative results of the semi-structured interviews are presented in narrative synthesis. Data of the different methods were triangulated, seeking to integrate the perspective of managers, professionals and families, in addition to performing a comparative analysis between the cases. Quantitative data were encoded and computed in Epidata, and Epidata Stat was used for descriptive analysis.

The project was approved by the Research Ethics Committee of Faculdade Maria Milza (Opinion 323/2011) and authorized by the Municipal Health Department.

## RESULTS

The results were organized according to dimensions and indicators featured in Table 1, which together represent strategies to achieve better care coordination based on strengthening PHC and integrating Healthcare Networks, based on the results of the cross-sectional study and interviews with managers in the health regions studied.

The organization of PHC as first contact of preference was one of the dimensions used to analyze how to strengthen coordination. The survey with families showed a high percentage of registered users who were aware of ESF, with the lowest rates in Vitória da Conquista. Many of them possibly did not use *Unidade de Saúde da Família* (USF – Family Health Unit), especially among teams with a high number of registered families. In the three municipalities, most users claimed to know where the USF facility was located and considered it easily accessible. The community health worker was known to respondents and a significant percentage reported receiving monthly home visits (Table 2).

**Table 2:** Primary Health Care as first contact of preference according to users and families registered with Family Health Teams (EqSF). Feira de Santana, Santo Antônio de Jesus e Vitória da Conquista, BA, Northeastern Brazil, 2013.

Indicator	Feira de Santana	Santo Antônio de Jesus	Vitória da Conquista
		
	%	%	%
Users aware of ESF (spontaneous + prompted)	97 (n = 588)	96 (n = 430)	73 (n = 572)
Users aware of ESF facility location	99 (n = 572)	96 (n = 412)	95 (n = 420)
Easy access to USF facility	97 (n = 565)	95 (n = 396)	97 (n = 399)
Users who reported accessing ESF facility on foot	95 (n = 565)	93 (n = 396)	87 (n = 399)
Users who are aware of CHW	98 (n = 572)	96 (n = 412)	96 (n = 420)
Users who have been visited by CHW	98 (n = 560)	92 (n = 395)	95 (n = 404)
Users visited by CHW at least once a month	68 (n = 546)	63 (n = 363)	68 (n = 386)
Families with private health insurance	22 (n = 588)	16 (n = 430)	19 (n = 572)
Family members covered by private health insurance	n = 129	n = 69	n = 106
One family member	46	43	44
Two family members	18	25	21
Three or more family members	17	32	35
Assessment of users regarding scheduling medical appointments*	n = 443	n = 308	n = 306
Very good/Good	68	58	54
Very poor/Poor	29	35	42
Assessment of users regarding obtaining medical appointments without previous scheduling – spontaneous demand*	n = 443	n = 308	n = 306
Very good/Good	42	39	29
Very poor/Poor	42	50	52
Health service sought for illnesses on weekdays	n = 588	n = 430	n = 572
Family Health Unit	43	49	39
Polyclinic	36	0	0
Public hospital outpatient/Emergency service	6	33	37
Clinic/Private hospital or emergency service	5	6	8
Private consultation	2	5	5
Other	8	7	11
Health service sought for illnesses on weekends/holidays/nighttime	n = 588	n = 430	n = 572
Family Health Unit	0.3	0	4
Polyclinic	71	0	0
Public hospital outpatient/Emergency service	13	83	68
Clinic/Private hospital or emergency service	6	6	10
Private consultation	1.7	4	4
Other	8	7	14

ESF: Family Health Strategy; USF: Family Health Unit; CHW: Community Health Worker

n = total number of respondents

* Users who reported having received care at a Family Health Unit in the previous 12 months.

The results also indicated significant search for other service modalities as first contact, even at times and days in which USF was open and in contexts of low private health insurance coverage. In Feira de Santana, polyclinics – emergency services operating 24 hours a day with specialists – strongly competed for first contact (Table 2). According to managers, the operation of polyclinics was quite varied: some of them provided initial care and referred users to USF, while others didn’t.

In Santo Antônio de Jesus, a regional hospital offering outpatient care by spontaneous demand, not linked to PHC, was routinely sought as first contact of preference, accounting for first contact services on weekdays, according to 33.0% of users (Table 2). For managers, hospital-centered culture was very strong, with spontaneous demand at the regional hospital consisting of users who firstly visited USF or sometimes didn’t even bother to go there, claiming there were no doctors or materials, either at the municipal center or other municipalities of the health region.

In Vitória da Conquista, the excessive number of people under the care of a single EqSF limited the capacity of professionals to meet needs in a resolvable and timely manner. This situation was synthesized by the manager: (...) *we see teams that are overcrowded with families*. (...). *We have been unable to ensure access even to those who seek it, so we have many accesses via emergency care of things that are not urgent.”* (Municipal Manager/Vitória da Conquista).

Another reason for users to seek different means of first contact, in the three municipalities, was poor or inexistent reception services, although professionals evaluated that the team incorporated care into meeting spontaneous demand and people seek USF first when they need care (Table 3). The evaluation of users indicates problems in scheduling medical appointments and, especially, in meeting spontaneous demand (Table 2), suggesting problems in the organization of first contact. Overall, the evaluations of professionals were more favorable regarding access conditions to USF, although the average waiting time for an appointment might exceed 15 days, according to 1/3 of professionals in Feira de Santana (Table 3).

**Table 3:** Primary Health Care (PHC) organization as first contact of preference, breadth and resolvability, provision of and access to specialized care, and communication between professionals according to physicians and nurses of Family Health Team (EqSF). Feira de Santana, Santo Antônio de Jesus e Vitória da Conquista, BA, Northeastern Brazil, 2013.

Indicator	Feira de Santana^a^	Santo Antônio de Jesus ^b^	Vitória da Conquista ^c^
		
	%	%	%
PHC organization as first contact of preference			
Completely agree/agree that the population first seeks USF for health care	92	77	91
Completely agree/agree that with ESF the population seeks fewer hospital and specialized services	81	74	84
Completely agree/agree that the population first seeks the hospital or emergency network for health care	19	10	19
Completely agree/agree that USF only provides care with previous scheduling	10	6	6
Completely agree/agree that USF provides care for spontaneous demand	88	97	98
Completely agree/agree that the average waiting time for a medical appointment at USF exceeds 15 days	33	16	19
PHC breadth and resolvability			
Evaluate as very satisfactory/Satisfactory the capacity of EqSF to solve 80% or more of cases treated at USF	89	77	78
Provision of sample collection for lab tests at USF	9	77	66
Evaluate as very satisfactory/Satisfactory the regular distribution of medicines by the team	69	84	75
Provision of and access to specialized care in Healthcare Network (HN)			
Most common pathway for patients from care at UBS to referral to specialized care			
The appointment is scheduled by UBS and the date is later informed to patient	60	48	94
The patient leaves UBS with a scheduled appointment	25	6	0
Patients are always/Most times able to schedule other services			
Appointment with specialists	75	61	63
Specialized test	76	55	75
Hospitalization	58	48	64
Communication between professionals			
Report completing medical record after each consultation	94	100	98
Always/Most times provide written information when patients are referred to other services	92	90	83
Always/Most times receive counter-referral following user’s consultation with a specialist	13	10	5
Completely agree/Agree that physicians are able to provide follow-up care for users of other health services	33	42	56
Completely agree/Agree that nurses are able to provide follow-up care for users of other health services	50	61	64

ESF: Family Health Strategy; USF: Family Health Unit; HN: Healthcare Networks; UBS: *Unidade Básica de Saúde* (Basic Health Unit)

^a^ 36 physicians and 70 nurses.

^b^ 10 physicians and 21 nurses.

^c^ 28 physicians and 36 nurses.

Although managers reported similar problems in consolidating PHC in the health region, strategies to face barriers and find solutions were still limited to the municipal level, being off the agenda of regional governance initiatives such as *Comissão Intergestores Regional* (Regional Inter-Managerial Commission). This was confirmed in the interviews, in which managers explained they could only account for PHC dynamics in their own municipality.

PHC breadth and resolvability are important dimensions of care coordination. In Feira de Santana, according to managers, these elements were compromised by factors such as: insufficient number of *Núcleos de Apoio à Saúde da Família* (NASF – Centers for Family Health Support), lack of oral health teams in all USF, reduced or simplified offer of health actions, few community interventions, low involvement of doctors in collective and administrative actions, and poor logistics and communications support for integration. Interviews indicated scarce and irregular supply of inputs and inadequate physical infrastructure and facilities in many USF.

In Vitória da Conquista, managers stressed the participation of professionals in offering technical and pedagogical support to EqSF as an alternative to the physician-centered view. It was emphasized that this is a recent experience in the health region, with insufficient numbers of NASF to meet the demands of EqSF, similar to the reality of Feira de Santana.

In the three regions, according to managers, attraction and retention of physicians generated distortions, influencing care quality and resolvability. Managers mentioned they were unable to select professionals with the necessary profile to work in ESF, and frequently “turned a blind eye” to absences and carelessness, with physicians often being “auctioned” among the region’s cities. However, they reported no effort to seek regional solutions to the problem.

Nonetheless, the results of the present study showed that physicians were responsible for the care of most users seeking USF. When asked about satisfaction concerning consultations with physicians, the majority (> 69%) reported being “very satisfied/satisfied” (Table 4).

**Table 4:** Primary Health Care breadth and resolvability according to users and families registered with Family Health Team (EqSF). Feira de Santana, Santo Antônio de Jesus e Vitória da Conquista, BA, Northeastern Brazil, 2013.

Indicator	Feira de Santana	Santo Antônio de Jesus	Vitória da Conquista
		
	%	%	%
EqSF professional responsible for care^a^	n = 541	n = 391	n = 397
Physician	90	83	88
Nurse	8	13	8
Other/Doesn’t know/Did not answer	2	4	4
Satisfaction with care by physician	n = 486	n = 323	n = 350
Very satisfied/Satisfied	81	69	83
Fair	15	21	13
Very dissatisfied/Dissatisfied	4	10	3
Doesn’t know/Did not answer	0	0	1
Health problem solved by EqSF care^a^	n = 541	n = 391	n = 397
Yes	83	70	78
No	17	30	22
Action taken when health problem is not solved	n = 91	n = 117	n = 87
Referral to public network specialist by EqSF	25	20	29
Direct search for care in public hospitals/emergency services	37	30	26
Direct search for care in private health network	23	33	24
No action taken/No search for health services	6	14	8
Other/Doesn’t know/Did not answer	9	3	13
Performance of clinical pathology tests requested by EqSF professional^b^	n = 280	n = 141	n = 207
All tests performed in the public network	58	52	67
Some tests performed in the public network	16	26	19
All tests performed in the private network	26	22	14
Access to medicines prescribed by EqSF^c^	n = 461	n = 341	n = 360
Received all medicines	40	43	31
Received some medicines	53	50	64
Did not receive any medicines	7	7	5

n = Total number of respondents

^a^ Users who reported having received care at a Family Health Unit.

^b^ Families that reported having done clinical pathology tests requested by Family Health Team (EqSF) in the previous 12 months.

^c^ Families that reported having needed medicines prescribed by Family Health Team (EqSF) in the previous 12 months.

Users in the three municipalities reported positively on having their problem resolved at USF (> 70%) (Table 4), similar to the evaluation by professionals (Table 3).

The survey with families indicated that even when they could not access a service or have their problems solved at ESF, in all three cases most people (50% to 62%) sought public services. However, an important percentage of the population sought care from the private sector (23% to 33%) (Table 4).

In Feira de Santana, samples for lab tests were not collected at USF, which was confirmed by professionals (Table 3). In turn, polyclinics collected biological samples, which prompted demand for their services. In Santo Antônio de Jesus and Vitória da Conquista, according to managers, test samples were collected at USF, including rural areas.

Among families that required lab tests requested by EqSF in the previous 12 months, 76%, 85% and 92%, respectively, in Santo Antônio de Jesus, Feira de Santana and Vitória da Conquista, reported being able to do them; however, between 52% and 67% of that total reported that all tests were done in the public network, with the best service offered in Vitória da Conquista. Between 14% and 26% of users reported doing tests in the private network, and about 1/4 were unable to do the prescribed test, especially in Feira de Santana (Table 4).

Among users served by EqSF, 85% to 91% required medicines in the three cases studied. Most (50% to 64%) reported receiving only a few (Table 4), but a significant percentage, around 40% in Santo Antônio de Jesus and Feira de Santana, received all medicines from SUS. Professionals, especially those working in Santo Antônio de Jesus, evaluated positively the regular distribution of medicines (Table 3).

Regarding the provision of and access to specialized care in Healthcare Networks, an essential dimension of coordination, in the three municipal centers of the health regions, managers pointed out that provision of procedures for therapeutic support and financial resources for their expansion were insufficient. Therefore, they had to cope with the short supply, compromising health requirements and generating ethical conflicts between the EqSF, since they had to, among the numerous needs, choose which users had “priority.”

Access to more complex technological services, in all three cases, mainly occurred via referral by PHC, according to managers. When the procedure was scheduled, the referral form usually returned to EqSF; therefore, users then returned to USF or were contacted by the community health worker (Tables 3 and 5). In Santo Antônio de Jesus, a significant percentage (29%) contacted the scheduling center directly (Table 5).

**Table 5:** Provision of and access to specialized care in Healthcare Networks and communication between professionals according to registered users and families. Feira de Santana, Santo Antônio de Jesus e Vitória da Conquista, BA, Northeastern Brazil, 2013.

Indicator	Feira de Santana	Santo Antônio de Jesus	Vitória da Conquista
		
	%	%	%
Reported having seen a specialist in the previous 12 months	51 (n = 572)	51.5 (n = 412)	55 (n = 420)
Service providing consultation with specialist	n = 292	n = 212	n = 232
Polyclinic	17	17	18
Public hospital outpatient care	15	9	14
Public hospital	12	16	18
Private health service (doctor’s office/hospital)	45	51	44
Other	11	7	6
Specialized appointment referred by EqSF	47 (n = 292)	31 (n = 212)	40 (n = 232)
Forms of access to specialized appointment referred by EqSF professional*	n = 136	n = 65	n = 92
Scheduled by USF and date and time informed later	64	46	71
Scheduled by USF and date and time informed immediately	4	2	13
Referral and appointment information received directly at Scheduling Center	0	29	4
Referral received, but health service sought independently	7	6	1
Referral received, but health service sought and paid for independently	11	9	3
Other	14	8	8
Reported having needed to do a specialized test requested by EqSF in the previous 12 months	43 (n = 572)	31 (n = 412)	33 (n = 420)
Performance of specialized test requested by EqSF	83 (n = 248)	59 (n = 126)	81 (n = 137)
Forms of access to specialized test	n = 206	n = 74	n = 111
Scheduled by USF and date and time informed later	69	34	75
Scheduled by USF and date and time informed immediately	3	5	10
Referral and appointment information received directly at Scheduling Center	0	24	1
Referral received, but health service sought and paid for independently	16	18	2
Other/Unable to inform	12	19	12
Families with hospitalization in the previous 12 months that received written post-discharge information for EqSF	24 (n = 78)	43 (n = 67)	17.5 (n = 80)
Families with hospitalization in the previous 12 months who reported requesting information or visit by a EqSF professional during hospitalization	35 (n = 78)	40 (n = 67)	27.5 (n = 80)
Professional responsible for seeking information or visiting patient during hospitalization – CHW	63 (n = 27)	67 (n = 27)	73 (n = 22)

EqSF: *Equipe de Saúde da Família* (Family Health Team); USF: Family Health Unit; HN: Healthcare Networks; CHW: Community Health Worker

n = total number of potential respondents.

* Users referred by EqSF who reported having seen a specialist.

In the three cases studied, most specialists were professionals with experience in the private sector. In Feira de Santana, there was no specialties center concentrating services, and therefore specialists hired by public notice could work in their private clinics, basic health units or polyclinics. In Santo Antônio de Jesus and Vitória da Conquista, specialized services were hired by public tender, with common non-attendance of providers.

Besides the provision of care to the municipal center, services were procured to meet the needs of the health region’s *Programação Pactuada e Integrada* (Agreed and Integrated Program), which was not always possible based on regulated SUS prices. Often municipalities received a lower quota than agreed on, depending on what they managed to “negotiate” with private providers. For managers, the heavy reliance on the private sector represented an obstacle: “(...) *we live in a capitalist world, and must work with SUS, which is a completely non-capitalist model; making this system work depending on the private sector, that generates conflict”* (state manager).

The purchase of specialized services within the logic of procurement procedures was also mentioned by managers as a factor that undermines the possibility of building an integrated network and care coordination via PHC: “*It is fragmented from the start...*” (municipal manager/Santo Antônio de Jesus).

Queues and long waiting times for specialized care were common situations in all three cases. Requests for tests or procedures without protocols or well-defined criteria intensified problems in the regions. In Feira de Santana, private sector doctors could request “high complexity” tests, which were authorized by the scheduling center, competing with SUS users. Setting up task forces was a strategy to minimize waiting times in the three municipal centers.

For managers, expanding services was not sufficient, since there were no specialists available in the inland areas of Bahia. They emphasized that care gaps varied widely in the state, although advances were mentioned with the building of regional hospitals.

The difficulties in providing specialized care are reflected in the assessment of users. In all three cases, about half of those who were aware of ESF reported having needed to consult a specialist in the previous 12 months. Of those, 44% to 51% sought treatment in the private sector, commonly without referral by EqSF. In Feira de Santana and Santo Antônio de Jesus, 11% and 9% of users, respectively, sought private care, even with referral by EqSF (Table 5). About 2/3 of professionals reported they were able to schedule appointments with specialists “always or most times.” In all three cases, greater difficulties were observed in relation to scheduling hospitalization, according to professionals (Table 3). Only Vitória da Conquista had its own central service to regulate hospital beds and admissions.

Part of users who were aware of ESF (31% to 43%) reported the need for specialized tests in the previous 12 months. Of those who did the tests, most users in Feira de Santana (72%) and Vitória da Conquista (85%) stated that they were scheduled by EqSF.

The development of thematic networks (care lines) was seen as an advance in creating regionalized networks and overcoming fragmentation. Thematic networks would be a resource for creating a state plan for regional health care, according to state managers.

Regarding communication between professionals in the network, the last dimension of coordination to be analyzed, managers said the system of counter-referral was deficient, further worsened when services were provided by the contracted network. Counter-referral only occurred at the request or demand of the actual user. Referral was an instrument for scheduling procedures, and not of interprofessional communication. The survey with professionals showed that only 5% to 13% reported receiving counter-referrals frequently (Table 3).

Computerization of USF was partial and, if any, limited to scheduling appointments, and never used as a communication flow instrument. In Feira de Santana, the existence of electronic medical records was reported in some units, although they were not shared with the other network services. According to managers, implementation of clinical protocols was incipient.

In Santo Antônio de Jesus, about 43% of households reported receiving post-discharge information, and 40% reported requesting information or visits by EqSF professionals during hospitalization, especially community health workers (Table 5). For professionals, there were difficulties in follow-up care for users of services outside PHC (Table 3).

## DISCUSSION

The four dimensions investigated point to limits that endanger regional care coordinated by PHC. In this same perspective, Fausto et al.^8^ reaffirm that coordination by PHC depends on timely and resolvable first contact, attention to and care of spontaneous demand, comprehensive provision of services, and technical support. In the cases studied, the role of first contact of preference faces strong competition from hospital outpatient and emergency care services, outside the network, often devised to meet the demand for specialized care, such as regional hospitals. This situation is aggravated by problems of work organization in PHC, with disparity between programmed actions and spontaneous demand, barriers also identified by other studies^2^ ^,^ ^8^ ^,^ ^9^, and excessive number of users per EqSF.

There is need to expand clinical nursing actions, Centers for Family Health Support, and oral health as measures that could help people recognize that EqSF is not restricted to physicians. The study also points to the need to strengthen PHC by the regular provision of medicines and other inputs. Despite the constraints, PHC, in the perception of users and professionals, seems to have good resolvability, which reaffirms the need for investments to enhance it. The indistinct search for public services as first contact, whether USF, polyclinics or hospitals, demonstrates the need to organize and coordinate services offered by the network, so that access via PHC is preferred.

In all three cases, users expand their options as services are increasingly fragmented and coordination is dispersed. Cecilio et al.^7^ show that although users value USF, they multiply their possibilities by combining expected PHC resources with other services on the network. When coordination is extrapolated to health regions, the role of EqSF dissipates, for several reasons. Provision of specialized services is highly dependent on the private sector, whose public insufficiency and underfunding, combined with occasional inadequate use, can be identified as factors that hinder the establishment of regional networks. In addition, the selected health regions have historical care deficit, difficulty in attracting and offering certain specialties, physicians disinterested in the civil service (due to precarious labor relations in SUS), and high bargaining power of some specialties. Such problems go beyond the management capacity of isolated municipalities, requiring a shared stance^15^.

Although access to specialties via PHC in the health region has been formally organized, the incorporation of specialists by procurement of procedures accentuates fragmentation and weakens the mechanisms of care coordination and regulation. While large municipalities prioritize the establishment of local specialized services^1^, in the context of health regions there seems to be a pressing need for other ways of incorporating therapeutic support, in a perspective of intercity networks. In this sense, initiatives such as *Comissão Intergestores Regional* (Regional Inter-Managerial Commission) need to be strengthened and recognized as means of joint regional governance, able to overcome the municipal logic, which is ineffective to build regional networks for comprehensive care. To this end, Regional Intermanagers Commission (CIR) should bring together strategies for managers to establish joint health agreements to enhance the provision of public services, overcoming the fragile contract mechanisms with the private sector, since there is no proper monitoring of agreed goals among private providers, other than *post factum* auditing. In general terms, there is a relationship of mutual dependence between the public and private sectors; however, private contractors providing services to SUS have gained the upper hand, defining market prices for procedures and services to the detriment of public interest.

The need for communication between professionals and providers to achieve better care coordination is a consensus^5^ ^,^ ^13^ ^,^ ^a^. Coordination actions will likely fail under the sole responsibility of physicians^4^. In this study, for example, the community health worker was responsible for seeking information and visiting patients during hospitalization, indicating potential ways to maximize action horizontal coordination.

Unforeseen findings of this study, which nevertheless are relevant to the analyzed subject, include the absence of physicians at USF during data collection, carried out before and during the municipal elections. Physicians accounted for a greater percentage of losses, especially because of the absence or sporadic presence of these professionals at USF. Even though the election campaign may have generated instability and increased the precariousness of labor relations, especially in Feira de Santana and Santo Antônio de Jesus, turnover was higher among physicians, especially due to their greater employability and possibility of establishing new links in other municipalities. The losses are revealing of a concrete situation also experienced by users, influencing difficulty of access and timely care, leading to the search for emergency care services and, ultimately, thwarting care coordination by PHC.

The constraints of PHC coordination are varied, comprising a set of services that do not compose an integrated network with a view to coordinating subjects, knowledge and practices^3^, and lack of strong PHC essential attributes, requiring initiatives that go beyond municipal boundaries to organize the health care network within health regions.

Nevertheless, it is clear that the success of the health regions studied requires the fulfillment of responsibilities among managers of different entities who, despite the normative expectations of regionalization, are unable to agree on a plan capable of taking the health territory beyond a bureaucratic and programmatic vision. The three regions indicate that, regarding care coordination, there is urgent need for expansion and qualification of first contact services via PHC due to persisting problems of fragmentation and disorderly search for services provided without adequate care regulation.

The health regions indicate a shift from decentralization and a path to enable care integration^16^. However, this study shows that coordination, when it occurs, is limited to the municipal centers of the health regions, i.e., as the vast majority of municipalities need services offered by other entities, care coordination via PHC becomes unfeasible, losing itself in the bureaucratic flow of scheduling centers. Thus, even if a wide offer of services is achieved in a given territory, coordination is an essential attribute to enable continuous care and integrated services, requiring, more than ever, a strong PHC base.

## Footnotes

a Schang L, Waibel S, Thomson S. Measuring care coordination: health system and patient perspectives: report prepared for the Main Association of Austrian Social Security Institutions. London: LSE Health; 2013 [cited 2015 Jul 5]. Available from: http://eprints.lse.ac.uk/59573/1/__lse.ac.uk_storage_LIBRARY_Secondary_libfile_shared_repository_Content_Schang%2C%20L_Schang_Measuring%20care%20coordination_2014.pdf

b Ministério da Saúde (BR). Portaria 2.488, de 21 de outubro de 2011. Aprova a Política Nacional de Atenção Básica, estabelecendo a revisão de diretrizes e normas para a organização da Atenção Básica, para a Estratégia Saúde da Família (ESF) e o Programa de Agentes Comunitários de Saúde (PACS). Brasília (DF); 2011 [cited 2015 Jul 5]. Available from: http://bvsms.saude.gov.br/bvs/saudelegis/gm/2011/prt2488_21_10_2011.html

c Ministério da Saúde (BR), Departamento de Atenção Básica. Histórico de cobertura da Saúde da Família. Brasília (DF); 2015 [cited 2015 nov 5]. Available from: http://dab.saude.gov.br/portaldab/historico_cobertura_sf.php

d Giovanella L, Moraes SME, Mendonça MHM. Estudo de caso sobre implementação da Estratégia Saúde da Família em quatro grandes centros urbanos: relatório final. Rio de Janeiro: Fiocruz; 2008.

e Ministério da Saúde (BR), Diretoria de Atenção Básica. Caderno de Avaliação e Monitoramento da Atenção Básica. Brasília (DF); 2012 [cited 2013 Oct 1]. Available from: http://www.saude.ba.gov.br/dab/index.php?option=com_content&id=450&Itemid=145
